# Effect of application time of 38% silver diamine fluoride solution on arresting early childhood caries in preschool children: a randomised double-blinded controlled trial protocol

**DOI:** 10.1186/s13063-022-06130-1

**Published:** 2022-03-15

**Authors:** Iliana Gehui Yan, Faith Miaomiao Zheng, Sherry Shiqian Gao, Duangporn Duangthip, Edward Chin Man Lo, Chun Hung Chu

**Affiliations:** 1 Faculty of Dentistry, The University of Hong Kong, Hong Kong, SAR China; 2 Department of Stomatology, School of Medicine, Xiamen University, Xiamen, Fujian Province China

## Abstract

**Background:**

To study the caries lesion activity response to topical 38% silver diamine fluoride (SDF) therapy with increasing treatment application time.

**Methods/design:**

The design is a stratified-randomised, double-blind, active-controlled, parallel-group clinical trial with nine treatment arms. The trial will involve recruiting at least 414 3- to 5-year-old kindergarten children with caries, who will receive approximately 0.004 mL of 38% SDF (the typical amount applied per the manufacturer’s instructions) to treat each caries lesion. The children will be stratified by caries status, randomised by blocks, and allocated to nine groups of SDF application times: 3, 5, 10, 15, 30, 45, 60, 120, and 180 s. The outcome measure is caries lesion activity (active/arrest) at the tooth-surface level at 6 months post-initial treatment. A calibrated dentist will conduct the blinded clinical examinations at baseline and at the 6-month follow-up. In addition, the parents will be surveyed to examine the effects of the moderating variables, such as oral hygiene, on caries lesion activity. The hypothesis is that a monotonically increasing trend can be found between the SDF application time and the proportion of caries lesions that are arrested. The Cochran-Armitage test for trends in proportions, corrected for clustering within children, will be used to determine the relationship between the exposure to SDF (the SDF application time) and the response (proportion of lesions arrested) in children, taking into consideration the effect of the moderating variables as well as the nesting of multiple caries lesions within an individual child. An EC 80 analysis (an 80% maximal concentration) will be used to determine the exposure (the SDF application time) for 80% caries lesion arrest. Bootstrap methods will be used for clustered data and will be resampled by clustering to determine the 95% confidence interval.

**Discussion:**

This study will help with determining the optimal application time for SDF treatment. It will provide an evidence-based protocol for the use of SDF to arrest tooth decay in the primary teeth of young children. The results will inform an evidence-based SDF protocol to arrest caries, which affects 573 million children with tooth decay worldwide.

**Trial registration:**

ClinicalTrials.gov NCT04655430. Registered on 7th December 2020.

**Supplementary Information:**

The online version contains supplementary material available at 10.1186/s13063-022-06130-1.

## Background

Early childhood caries (ECC), which causes pain and infection, remains a challenging problem [1]. Untreated caries will progress into the tooth pulp to eventually form a dental abscess [2]. In addition, premature tooth loss due to ECC affects oral function and dentition. Poor dentition significantly affects children’s nutrition and consequently their growth, development, and general health. ECC is particularly prevalent among socially disadvantaged children, such as those from poor families and those whose parents have low educational levels [3, 4]. However, prevailing restorative methods for preventing and treating ECC are neither available nor affordable for these children [5].

Clinical studies have demonstrated that using 38% silver diamine fluoride (SDF) prevents and arrests ECC [6] A literature review of SDF suggested that 38% SDF can be an effective agent in preventing new caries as well as arresting dental caries in the primary teeth of children [5]. Another review also concluded that SDF is an effective, efficient, and equitable caries-control agent [7]. Milgrom and Chi (2011) advocated for SDF therapy as an important prevention-centred caries management strategy during critical early childhood periods [8].

Clinicians typically apply 38% SDF solution topically to a dental caries lesion and then leave it on the tooth for a certain amount of time before washing away any excess. Studies have used arbitrary application times with no consensus. However, clinicians have recommended a range of increasing application times with no scientific evidence (Table 1). A short application time is desirable because SDF is frequently applied to very young children who have difficulty with cooperating. Often caries can affect multiple teeth, which makes SDF application even more difficult, especially in younger children. A lack of cooperation often compromises treatment or renders SDF treatment impossible or ineffective. A search found no published or registered trials on the SDF exposure time-response relationship. Thus, this study addresses an important gap in the knowledge regarding SDF treatment.

**Table 1:** Recommendations for application protocol of silver diamine fluoride (SDF) solution

Authors, year, reference	SDF application protocol
Horst et al. 2016 *Journal of California Dental Association* 44:16–28.	Isolate the tooth with gauze Apply SDF to the caries of the tooth, and leave it for up to **60 s** Rinse with water
Crystal et al. 2017 *Pediatric Dentistry* 39:E135-E145.	Isolate the tooth with cotton rolls and/or gauze Apply SDF to the caries of the tooth, and leave it for **60 s** Dry excess SDF with a gentle flow of compressed air
Croll & Berg 2018 *Inside Dental Hygiene Continuing Education Course* (https://idh.cdeworld.com/courses/5155)	Blot-dry the surface of the carious lesion with a cotton swab and an air syringe Paint on SDF, and continue to dab it; keep lesion isolated for **60 s** Blot with cotton swab, and then, cover it with fluoride varnish
American Dental Association 2018 (https://www.youtube.com/watch?v=a0HH7GifdM4)	Clear and dry the caries of the tooth with cotton pellet and/or gauze Apply SDF to the caries of the tooth, and leave it for **60 s** Dry excess SDF with gauze if necessary
Yee et al. 2009 *Journal of Dental Research* 88:644-647.	Isolate the tooth with cotton rolls Apply SDF to the caries of the tooth, and leave it for **120 s** Remove excess via gentle blotting with cotton pellet
Clemens et al. 2018 *Journal of Public Health Dentistry* 78:63-68.	Isolate the tooth with cotton rolls and/or gauze The exposure time of SDF treatment is aimed at **120** s Remove excess SDF with gauze, and then, apply fluoride varnish
Seifo et al. 2020 *British Dental Journal* 228:75-81.	Isolate the tooth with cotton rolls Apply SDF to the caries of the tooth, and leave it for **120 s** Remove excess via gentle blotting with cotton pellet
Llodra et al. 2005 *Journal of Dental Research* 84:721-724.	Isolate the tooth with cotton rolls Apply SDF to the caries of the tooth, and leave it for **180 s** Wash with a 30s water spray three minutes after the application
Burgette et al. 2019 *Journal of Dentistry for Children* 86:32-39.	Isolate the tooth with cotton rolls and/or gauze Apply SDF to the caries of the tooth, and leave it for **180 s** Dry excess SDF, and then, apply fluoride varnish

### Aim

To determine the proportion of caries lesions arrested by topical 38% SDF therapy with increasing treatment application times.

### Objectives

The primary objective is to study the proportion of caries lesions arrested by topical 38% SDF therapy with increasing treatment application times. The secondary objective is to study staining effect (discolouration) of the caries lesion with increasing treatment application times of topical 38% SDF therapy.

### Hypothesis

A monotonically increasing trend can be found between the SDF application time and the proportion of caries lesions arrested and intensity of discolouration of carious lesion.

## Methods/design

### Trial design

**Fig. 1:**
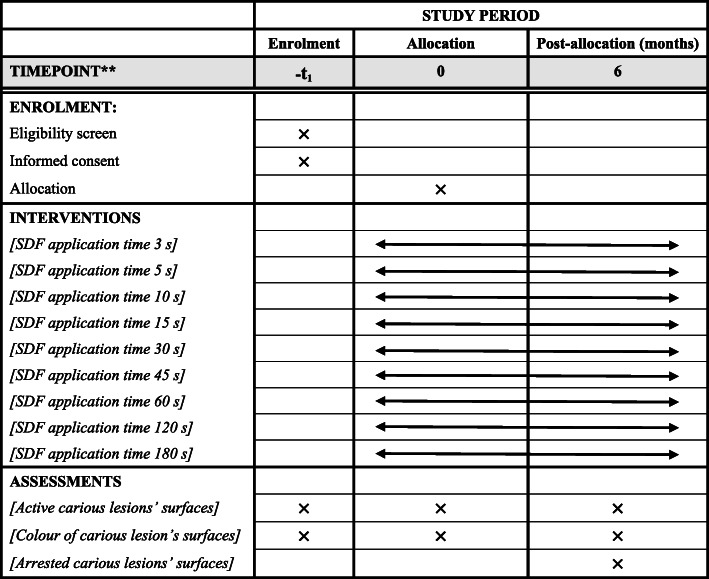
Schedule of enrolment, intervention, and assessments of application time of 38% silver diamine fluoride (SDF) solution to arrest early childhood caries in preschool children

This is a stratified-randomised, double-blind, active-controlled, parallel-group clinical trial with eight arms. The extension of the Consolidated Standards of Reporting Trials 2010 Statement will be followed [9]. In addition, the design and report of this protocol follow the Standard Protocol Items: Recommendations for Interventional Trials statement (Additional file 1. SPIRIT 2013 Checklist) [10]. The schedule of this study is shown in Fig. 1.

### Setting

Kindergartens in Hong Kong that have joined our outreach dental service will be invited to take part in this study. An invitation letter will be sent to the principals of kindergartens explaining the study’s purpose and procedures (Additional file 2). Any preschool children who have tooth decay will be invited to join the study. Written informed consent explaining the study’s purpose and procedures will be sought from their parents. A trained dentist and a dental assistant will go to the kindergartens to assess the caries-arresting effects of SDF with different application times on preschool children.

### Participants

The children included in the study should (a) be aged 3–5 years and attending kindergarten, (b) be generally healthy, (c) have parental consent, and (d) have untreated caries that extends into the dentine. Children who are uncooperative and difficult to manage, have major systemic diseases, or are on long-term medications will be excluded. If the participated child does not return for the follow-up examination, a project assistant will contact the parent and ask for will the reasons for drop out.

### Recruitment and screening

The University of Hong Kong is providing outreach dental care service to all 180,000 kindergarten children in Hong Kong (https://www.jccohp.hku.hk/). A research assistant will invite sufficient number of kindergartens for achieving adequate participant enrolment to reach the target sample size. A trained project assistant will explain the objectives and procedures of this study to the teachers of the participating kindergartens. The teachers will distribute an invitation letter and an informed written consent to the parents of children attending the first and second year of the kindergarten. The written consent will be collected from the parents before the trial takes place. Oral health education will be provided to the children in the participating kindergartens, followed by initial oral examinations. Trained field workers on the outreach team will recruit potential participants after the initial oral examinations.

### Clinical examination

The trained dentist will perform clinical examinations of the participating children in the kindergartens. The dentist will carry out a careful visual inspection with the aid of a World Health Organization (WHO) Community Periodontal Index (CPI) periodontal probe and a front-surface dental mirror with light-emitting diode intra-oral illumination [11]. The oral hygiene status will be measured using the visible plaque index (VPI). Both the buccal and the lingual surfaces of six index teeth (55, 51, 63, 71, 75, and 83) will be examined [12]. The presence or absence of visible plaque on the caries surface will also be recorded.

Tooth status (decayed, missing, filled surface [dmfs] score), tooth discolouration, and hyper-mobility will be recorded in the chart [13]. Teeth with carious lesions extending into the pulps, or signs suggesting that the teeth are non-vital, such as tooth discolouration, hypermobility, or abscesses, will not be included in this study. Dental caries will be diagnosed at the cavitation level. The carious lesion will be gently explored using a WHO CPI probe in the centre of the lesion [14]. Great care will be taken to avoid tooth damage during the probing. A carious lesion will be recorded as active if softness is detected, whereas if the dentine surface is hard to probe, it will be classified as an arrested caries [6, 12]. All surfaces (i.e., buccal, lingual, mesial, distal, and occlusal for the posterior teeth) of each tooth will be assessed. In addition, the colour of the carious lesion will be classified into one of four categories according to PANTONE colour plates (Table 2) placed next to the carious lesion [15]. The primary endpoint or outcome measure is active carious lesion at baseline that becomes arrested in the 6-month follow-up. The same dentist will serve as the examiner throughout the 6-month follow-up. Intra-examiner agreement on the plaque and caries assessment will be monitored and conducted in 10% of the children at each stage of the clinical trial.

**Table 2:**
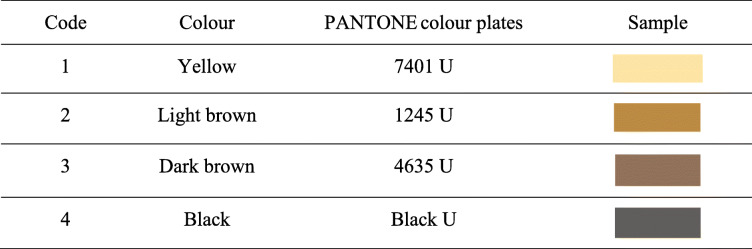
Coding for the colour assessment of dentine carious lesions

### Questionnaire survey

A validated parental questionnaire [3, 4] regarding children’s oral hygiene habits (daily tooth brushing frequency), parent-assisted tooth brushing, the main caretaker of the child, the use of toothpaste, the use of a bottle for feeding, bottle feeding before going to bed without brushing, daily snacking frequency, dental visits, the parental educational level, and the family’s total income will be administered at baseline. The questionnaire will also assess parental satisfaction with the child’s oral health and dental aesthetics. Another questionnaire survey will be conducted after 6 months.

### Randomisation and treatment allocation

**Fig. 2:**
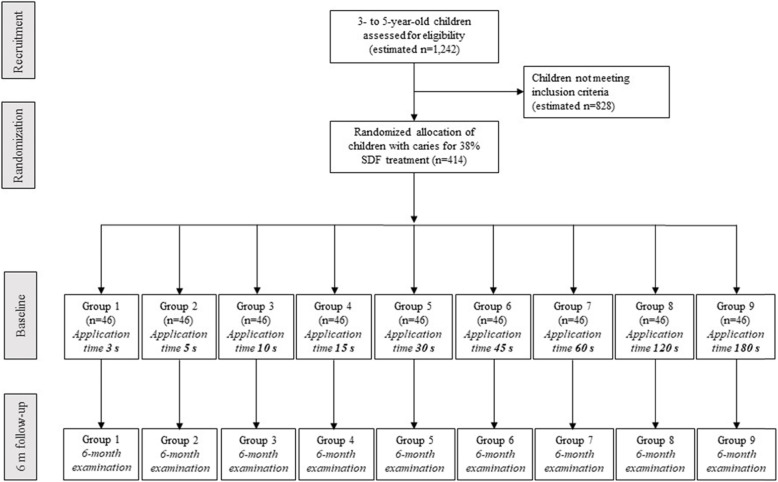
Flow diagram of the randomised clinical trial of the effects of 38% silver diamine fluoride (SDF) application time on arresting early childhood caries

The participating children with caries will first be categorised as either (1) having a high caries rate (or severe early childhood caries as defined by the American Academy of Paediatric Dentistry, 2008), which is defined as the presence of more than three untreated caries surfaces, or (2) having a low caries rate [16]. The children will be allocated via a stratified randomisation method with a block size of 18 into nine groups for the application of 38% SDF (Advantage Arrest Silver Diamine Fluoride 38%, Elevate Oral Care, FL, USA) on each carious lesion (Fig. 2):

Group 1 – A 3-s application

Group 2 – A 5-s application

Group 3 – A 10-s application

Group 4 – A 15-s application

Group 5 – A 30-s application

Group 6 – A 45-s application

Group 7 – A 60-s application

Group 8 – A 120-s application

Group 9 – A 180-s application

### Allocation concealment and implementation

The randomisation sequence will be generated by a computer by the statistician, who will maintain the random number sequence in opaque sealed envelopes to conceal the allocation sequence until the interventions are assigned. A dental assistant will open the envelope and assign the children to the treatment group according to the random number generated.

### Blinding

In this double-blind randomised clinical trial, a dental assistant will conduct the group allocation. The examiner and the child will not be informed of the treatment group allocation. Another operator will apply the SDF solution after the oral examination.

### Harms

A 24-h mobile contact number will be given to the parents of each child to call if there are any problems.

### Interventions

An independent operator will use a micro-brush (Advantage Arrest Applicators, Regular size, Elevate Oral Care, FL, US) to apply the 38% SDF solution. Each carious lesion will receive a single application of 38% SDF at different application times according to the allocated group. The amount of 38% SDF solution to be applied is approximately 0.004 mL [17]. The children will be instructed not to eat or drink for an hour after the SDF application. The carious surfaces will become hard and black if the treatment has successfully arrested the carious lesions. In case any harm occurs, the principal investigator will monitor the child and report the incident to the institutional review board (IRB). After the baseline examination, the research assistant will provide a report on the child’s oral health status to the parents. The report will feature a note asking the parents to report to the dentist if they notice any side effects on the treated tooth and the surrounding gingival tissue after SDF treatment.

### Follow-up evaluation

The follow-up oral examinations will be conducted 6 months later in the kindergartens. The same examiner will perform the examinations using the same equipment, procedure, and diagnostic criteria as those used in the baseline examinations. Each child’s oral hygiene and caries status will be recorded. In addition, the presence of visible plaque and the statuses of the included carious surfaces will be assessed. The colour of the arrested caries lesion will be classified and recorded as well [15]. The dentist will also record any sign of a tooth’s non-vitality, such as tooth hypermobility and an abscess. The examiner will not serve as the operator for the fluoride application.

### Data management and access to data

The statistician and co-investigators will work closely with the principal investigator to monitor trial conduct and safety, assess risks and benefits, and make recommendations to protect the participants of this clinical trial. Two independent research assistants will enter and compare the collected data to minimise data entry errors. The statistician will oversee the data entry, checking, and analysis. The investigators, the research assistant and the statistician have access to the data.

### Auditing

An independent auditor will conduct a systematic and independent examination of the trial-related activities and documents. The auditor will check the informed consent forms, documentation of the consent process and reported data to ensure protocol compliance.

### Protocol amendments

The principal investigators will report any amendment of the trial protocol or changes of the eligibility criteria, outcomes or analyses to the local institutional review board and to the trial registry.

### Outcome measure

The outcome measure (primary endpoint) is the carious lesion’s activity (active/arrested) at the tooth-surface level at 6 months post initial treatment. The outcome measure will be administered at baseline and at the 6-month follow-up examination. The children and their parents have the right to receive dental treatment from other dentists. Based on our knowledge and experience in the previous clinical trials [13, 14, 18], other dental personnel generally will not care for the participating children, and we will include questions about the receipt of other dental treatments at baseline and during the follow-up interviews.

### Post-trial care

All children will be under care through the outreach kindergarten service. Children who experience any side effects from the SDF application will be followed up by the outreach dentist.

### Sample size and power calculation

The sample size is estimated based on a caries arrest rate of 70% [19] for a 60s application. We assume that a monotonically increasing trend exists and that the caries arrest rate is 80% for a 180 s application. Therefore, the expected caries arrest rates are 65.25%, 65.42%, 65.83%, 66.25%, 67.50%, 68.75%, 70.00%, 75.00%, and 80.00% for 3-s, 5-s, 10-s, 15-s, 30-s, 45-s, 60-s, 120-s, and 180-s applications, respectively. The power will be set at 90%, and the one-sided type I error (α) will be set at 0.025 (equivalent to a two-sided type I error of α = 0.05). The required sample size for detecting proportion trends is 104 surfaces of carious lesions per group [20]. In this study, the mean number of surfaces of carious lesions at baseline per child is estimated to be 4.75 [14]. In addition, the anticipated intraclass correlation coefficient is 0.23. This clinical trial requires 195 surfaces of carious lesions from 41 children per group following the equation for a multi-level study [21]. We assume that the dropout rate is 0.5% and the non-compliance rate is 10%. At least 46 children per group, or a total of 414 children at baseline, need to be recruited for this randomised clinical trial.

### Statistical methods

We have developed a data management and statistical analysis protocol for the clinical trial. The protocol details the procedures for data entry, management, cleaning, and analysis. Two research assistants will enter the collected data into an Excel file, and they will compare the entered data to minimise data entry errors. The research assistants will also assess the intra-examiner agreement regarding caries diagnoses and the VPI at each time point using Cohen’s kappa statistics. The level of statistical significance for all two-sided tests will be set at 0.05.

We will perform the Cochran-Armitage test for trends in proportions with correction for clustering. In addition, we will explore the trend in the arrest rates of carious lesions with increasing SDF exposure (application) times, taking into consideration the effect of the moderating variables and the number of surfaces of the carious lesions. The power calculations will be based on a steady linear increase, and the power of the study will be less if a plateau is reached before 180 s. Thus, we will set the power at 90% in our sample size calculation and estimate the profile non-parametrically to see if any plateau is reached at a given time. EC80 (an 80% maximal concentration) analysis will be used to determine the exposure (the SDF application time) at which 80% of the carious lesions are arrested. Because individual children may have more than one lesion, we will use bootstrap methods for clustered data, and resample by cluster to determine the 95% confidence interval. An in-house statistician will perform statistical analyses using the software of IBM SPSS Statistics for Windows version 26 (IBM Corp. Armonk, NY, USA) and SAS OnDemand for Academics (SAS Institute, Inc., Cary, NC, USA).

This clinical trial will employ an intention-to-treat analysis to address dropouts. We will perform a sensitivity analysis (attributable estimand) to address the outcomes for those children not completing the 6-month study. We will assume that none of the treated teeth will be arrested among dropouts and thus will include dropouts in the sensitivity analysis. However, our experience from previous trials suggests that the number of children who will drop out in 6 months will likely be less than 1% [14, 18, 22].

### Dissemination

The investigators will communicate and report the trial results to participating parents, kindergarten teachers, researchers, healthcare professionals and the public through presentation in meetings and conferences. In addition, they will prepare abstracts and articles for publication in open access international journals.

### Ethical considerations

The IRB of the University of Hong Kong (HKU)/ Hospital Authority Hong Kong West (HAHKW) Cluster Ethics performed a full review and approved this clinical trial (IRB UW 20-737). Informed consent will be sought from the parents of each participating child prior to his or her participation in the trial. The trial will generally pose minimal risk to the participating children. However, the principal investigator will provide training to the field workers to minimise risk. The principal investigator will use the monitoring system and report regularly to the HKU/HAHKW IRB. In addition, we have a protocol for the management of side effects and we will report all side effects to the HKU/HAHKW IRB within 48 h. In addition, we will submit reports regularly to the HKU/HAHKW IRB for the external control of quality. An un-blinded member of the team will review the data on a continuous basis from the baseline and 6-month follow-up examinations to check their quality. The trial statistician will review the data at key points of the trial.

## Discussion

SDF therapy is a simple, non-invasive, and efficient nonsurgical treatment for young children [5]. It is well accepted among both children and clinicians for caries management [23]. In addition, it has great potential to aid the dental public health community in addressing dental caries in at-risk populations [24]. A review reported that SDF can arrest caries when it is applied to caries for 10 s to 180 s [25]. The manufacturer recommends a 30-s to 60-s application time. Most clinical guidelines recommend that SDF should be applied and retained on the teeth for 60 s to 180 s. However, scientific evidence supporting the recommendations is lacking. The optimal SDF application time in clinical practice remains unknown and thus requires further validation. Thus, this study will help with determining the optimal application time in SDF treatment. It will provide an evidence-based protocol for the use of SDF to arrest ECC. The results will inform an evidence-based SDF protocol for arresting the caries of many young children with tooth decay worldwide.

This trial will use the kappa statistic because it is a simple and frequently used statistical method for testing intra-examiner reliability. The intra-examiner reliability is important because it represents the extent to which the variables are appropriately rated in the trial. Cohen kappa is the corresponding reliability coefficient [26]. A limitation of the kappa statistic is that it is sensitive to the true prevalence of the variable measured. A high (or low) true prevalence will increase the expected agreement by chance. The Cochran-Armitage trend test will be used in this trial whether or not a linear trend is found when the response is binary [27]. This statistical method can be used to analyse ordinal data. The SDF application time can be ordered from 3 s to 180 s, and we expect that the caries arrest rate will increase when the SDF application time increases. The power of the study will be less if a plateau is reached before 180 s. Therefore, we set the power at 90% in our sample size calculation.

This trial will use EC_80_ (80% maximal concentration) analysis. EC_80_ can be defined as the exposure (the SDF application time) required to obtain 80% of caries arrest. We will use the bootstrap method for clustered data, and perform resample by cluster to determine the 95% confidence interval. The bootstrap method uses simulation to calculate standard errors, confidence intervals, and significance tests, and it does not rely on many assumptions [28]. It resamples (with replacement) the sample data and creates a large number of phantom samples known as bootstrap samples. A major advantage of using the bootstrap method is its simplicity. It is a straightforward way of deriving estimates of standard errors and confidence intervals for complex estimators of the distribution. However, this method relies on a representative sample and can be time-consuming. This clinical trial will also employ an intention-to-treat analysis to address dropouts. Intention-to-treat analysis is a method for analysing the results of all children according to the groups to which they are assigned, regardless of what application times they received. This method generates an unbiased conclusion regarding the effectiveness of the intervention [29].

## Trial status

This clinical trial was registered at ClinicalTrial.gov (USA), and the registration number is NCT04655430. The date of registration is on 30 November 2020. This protocol manuscript was developed on 28 August 2021. The recruitment of the participants will start on 1 October 2021 and is expected to be completed by 31 December 2022.

## Supplementary Information

**Additional file 1:.** SPIRIT 2013 Checklist

**Additional file 2:.** Consent form

## Data Availability

The research team will share the results of the dental examination of the participating children with their parents via individual oral health report. The team will share the results of the study with academia via publications and presentations. The datasets generated in this trial will be available from the primary investigator on a legitimate request.
