# Supplementary material for: Effect of application time of 38% silver diamine fluoride solution on arresting early childhood caries in preschool children: a randomised double-blinded controlled trial protocol
Source: Trials. 2022 Mar 15;23:215. doi: 10.1186/s13063-022-06130-1 (PMC8922752; doi:10.1186/s13063-022-06130-1)
Supplement: Supplementary file 2 — Additional file 2:. Consent form [file 13063_2022_6130_MOESM2_ESM.pdf]

2nd July 2021

Dear Parents of preschool children,

Re: Effect of application time of 38% silver diamine fluoride solution on arresting early childhood caries in preschool children: a randomized double blinded controlled trial

The Faculty of Dentistry of the University of Hong Kong (HKU) is now conducting a clinical study about Effect of application time of 38% silver diamine fluoride solution on arresting early childhood caries in preschool children: a randomized double blinded controlled trial.

The details of the activity would be as follows:

Target: K1 and K2 children;

Proposed period: Start in October 2021;

Activities:

(1) Questionnaire survey: Parents will fill in a two-page questionnaire about their children's daily oral hygiene habits and demographic background;
(2) Oral examination: Basic oral examination for school children will be carried out by a registered dentist;
(3) Treatment: If the children have not decayed tooth, they do not need to receive SDF treatment, and follow-up for one year. If the children have decayed tooth, they will be treated with SDF at different time. We will provide check-up and SDF treatment on a regular oral examination every six months in the following year

Your child's kindergarten is one of the participating kindergartens. If you choose to participate, we will provide a free dental examination to your child in the kindergarten by a dentist. These activities will be carried out in your child's kindergarten. The whole procedure will take 5 minutes. No radiographs will be taken. No lifestyle or dietary restriction is required during the study period. Each participating child will receive a brief report regarding his/her dental health status after the examination, so that parents can have a better understanding of their children's oral development and hygiene, and deal with their children's dental problems as soon as possible.Those who need any other dental services such as extraction can be treated by their own dentist at their own cost.

SDF treatment is not only safe, painless, rapid and easy to be accepted by children, but also has been shown to effectively arrest dental decay.If there is tooth decay presented, the decayed lesion will become harden and darken after the fluoride treatment. Please be noted that the black stain means the tooth decay has been successfully stopped from progressing. The black stain will not fade away until the replacement of milk teeth. However, if there is a layer of bacteria presented on the teeth, the fluoride may kill the bacteria and stain the bacteria black. Staining on bacteria is not permanent. It can be moved by professional toothbrushing. SDF may stain grey if accidentally dropped onto skin, but it will disappear within one or two week's time.

Your children's teeth (not face) will be photographed for the research and academic purposes. We cannot record and use images of children unless the form has been signed by a parent or guardian. Participation in this issue is completely voluntary. You can choose not to let your child participate or to withdraw at any time after enrolment without any detriment.

It is up to you to decide whether or not to let your child to take part. If you decide to take part in the study, please fill in the questionnaire attached as well as authorise us to provide a dental examination and fluoride treatment to your children in school time. You are still free to

withdraw at any time and without giving a reason. This will not affect the standard of care you receive in the future.

You have the rights of access to personal data and publicly available study results, if and when needed. Under the laws of Hong Kong (in particular the Personal Data (Privacy) Ordinance, Cap 486), you enjoy or may enjoy rights for the protection of the confidentiality of your personal data, such as those regarding the collection, custody, retention, management, control, use (including analysis or comparison), transfer in or out of Hong Kong, non-disclosure, erasure and/or in any way dealing with or disposing of any of your personal data in or for this study. For any query, you should consult the Privacy Commissioner for Personal Data or his office (tel no. 2827 2827) as to the proper monitoring or supervision of your personal data protection so that your full awareness and understanding of the significance of compliance with the law governing privacy data is assured.

By consenting to participate in this study, you expressly authorise:

a) the principal investigator and his research team and the ethics committee (Institutional Review Board of the University of Hong Kong / Hospital Authority Hong Kong West Cluster) responsible for overseeing this study to get access to, to use, and to retain your personal data for the purposes and in the manner described in this informed consent process; and
b) the relevant government agencies (e.g. the Hong Kong Department of Health) to get access to your personal data for the purposes of checking and verifying the integrity of study data and assessing compliance with the study protocol and relevant requirements.

It is possible that when taking part in the oral examination, your child may feel discomfort. If your child is too unwilling to be checked, the oral examination will stop immediately. If your child is harmed by taking part in this study, there are no special compensation arrangements. If your child is harmed due to someone's negligence, then you may have grounds for a legal action. Regardless of this, if you wish to complain about any aspect of the way your child has been approached during the course of this study, the normal health service complaints mechanisms may be available to you.

After examination, each of the participants will receive a set of souvenir. We hope that the oral examination and fluoride treatment will help you to understand the oral health status of your child. This study has been reviewed by Institutional Review Board of the University of Hong Kong / Hospital Authority Hong Kong West Cluster. If you have any enquiry, please contact me at 2859 0287 during office hours. Please complete the following consent if you agree to participate in this study. Thank you very much.

This research project will be provided for free by the Faculty of Dentistry, HKU, with no commercial interest. We sincerely ask for your approval to allow your child to participate. You are entitled to refuse to participate in this research project and this will not affect your child's rights of medical and dental services. You can also withdraw from the research any time. All information and data collected will be kept confidential and use for research purpose only. If you have any questions, you are welcome to reach Prof. C.H. Chu by phone 28590287 or through e-mail chchu@hku.hk.

Thank you.

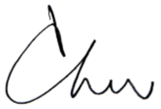

Prof. Chu Chun Hung, Clinical Professor,
Faculty of Dentistry, The University of Hong Kong

HKUDentistry
Faculty of Dentistry · The University of Hong Kong
香港大學牙醫學院

PATIENT/SUBJECT CONSENT FORM

Child name: ____________________

Title of Project: **Effect of application time of 38% silver diamine fluoride solution on arresting early childhood caries in preschool children: a randomized double blinded controlled trial**

Name of Researcher: Prof. Chu Chun Hung

Please tick ✓in the box

1. I confirm that I have read and understood the information sheet for the above study and have had the opportunity to ask questions. ☐
2. I understand that my participation is voluntary and that I am free to withdraw any time, without giving any reason, without my medical care or legal rights being affected. ☐
3. I understand that sections of any of my medical notes may be looked at by responsible individuals from Faculty of Dentistry, The University of Hong Kong or from Institutional Review Board of the University of Hong Kong / Hospital Authority Hong Kong West Cluster. I give permission for these individuals to have access to my records. ☐
4. Regarding this three-year study:

   4.1. I ☐ **agree** / ☐ **not agree** to complete the questionnaire,and let my child undergo oral examination and fluoride treatment;

   4.2. I ☐ **agree** / ☐ **not agree** to have photos taken of the teeth of my child (not including face).

____________________	____________________	____________________
Name of Parent / Guardian	Date	Signature
____________________	____________________	____________________
Researcher	Date	Signature
